# La valgisation tibiale par addition d'ouverture interne avec comblement cimenté dans la gonarthrose fémorotibiale médiale sur 38 genu-varum

**DOI:** 10.11604/pamj.2015.20.204.6065

**Published:** 2015-03-05

**Authors:** Mohamed Amine Mahraoui, Merouane Abouchane, Yassir El Andaloussi, Ahmed Reda Haddoun, Mohamed Nechad

**Affiliations:** 1 Centre Hospitalier Universitaire Ibn Rochd Casablanca, Casablanca, Maroc

**Keywords:** Ostéotomie, valgisation, tibia, addition, cimenté, Osteotomy, valgization, tibia, addition, cement

## Abstract

Le genu varum sur gonarthrose fémorotibiale interne est une pathologie en nette recrudescence dans notre pays, affectant spécialement les femmes âgées et hautement invalidante chez l'adulte jeune; Souvent, elle pose un problème d'indication et de choix thérapeutique d'ordre multifactoriel. L'ostéotomie tibiale de valgisation par ouverture interne est une technique de référence, bien connue depuis longtemps et d'efficacité validée à court, moyen et long terme, et constitue un outil thérapeutique de choix et d'apport marqué, notamment pour les sujets jeunes actifs avec gonarthrose débutante. Par ailleurs, cette technique peut voir ses indications s’élargir au dépend de l’âge et du stade évolutif. Le but de notre travail est d’évaluer les résultats anatomiques et fonctionnels de notre technique d'ostéotomie tibiale de valgisation avec comblement cimenté chez l'adulte jeune de plus de 40 ans et de préciser les facteurs pronostiques qui régissent ces résultats. Ce travail propose à travers une étude rétrospectivement menée à propos de 38 genoux opérés chez 28 patients de dresser un bilan épidémiologique, clinique, et radiologique afin d’évaluer les résultats anatomiques et fonctionnels immédiats et à distance avec un recul minimum de 2 ans, de l'ostéotomie tibiale de valgisation avec comblement cimenté. Les 28 cas ont été revus à un recul moyen de 3,7 ans avec des extrêmes entre 2 et 9 ans, l’âge moyen de nos malades était de 52 ans avec des extrêmes de 40 à 67 ans, le sexe féminin était prédominant (64%). Le genu varum était primitif dans 20 cas (71,4%), et secondaire dans 8 cas (28,5%). Les stades I et II d'Ahlback constituaient la majorité des cas de l'arthrose fémoro-tibiale (94,7%). La déviation angulaire globale moyenne était de 11,3° avec des extrêmes de 8,5° à 18°. Les résultats évalués selon le protocole du groupe Guépar étaient excellents et très bons dans 86% des cas, et moyens et mauvais dans 14% des cas. Les meilleurs résultats ont été notés avec un âge au moment de l'ostéotomie de 60 ans, une arthrose au stade I et II d'Ahlback et un varus initial moyen ne dépassant pas 15°. La normocorrection a permis d'obtenir de bons résultats. Les complications postopératoires étaient rares sans conséquence sur les résultats thérapeutiques.

## Introduction

Le genu varum se définit comme une déviation axiale du genou dans le plan frontal. La position du genou est alors en varus, le compartiment fémorotibial interne devient trop chargé alors que l'externe est déchargé. Il en résulte une dégradation cartilagineuse puis osseuse du compartiment fémorotibial interne qui augmente à son tour l'importance du varus. Cette déviation axiale peut être idiopathique constitutionnelle et familiale, comme elle peut être secondaire à une lésion post traumatique, fracture articulaire dystrophie résiduelle dans l'enfance, ménisectomie interne totale. Ainsi la prise en charge thérapeutique visant à restituer un genou normo-axé, à diminuer les contraintes excessives sur le compartiment fémorotibial interne et à empêcher l’évolution vers le stade ultime de l'arthrose comporte de multiples choix. Outre le traitement médical et les règles hygiénodiététiques, l'ostéotomie tibiale haute de valgisation a été proposée depuis longtemps comme un traitement conservateur de l'arthrose. Les deux techniques les plus répandues sont d'une part l'ostéotomie d'addition interne, et d'autre part, l'ostéotomie par soustraction externe. Lors de l'ostéotomie d'ouverture médiale, plusieurs types de cales peuvent être utilisés: Cale de ciment qui fait l'objet de notre étude, et qui remplacé depuis 1985 les greffons iliaques corticospongieux utilisés depuis longtemps mais dont le prélèvement est douloureux et alourdit la chirurgie; Cale par des allogreffes osseuses: cale d'alumine, substituts osseux. Le but de cette étude est d’évaluer à travers l'expérience de notre service, les résultats anatomiques et fonctionnels de l'ostéotomie tibiale haute de valgisation par addition interne d'une cale de ciment sur genu varum d′origine tibiale et d'en déduire les indications. L'ostéotomie tibiale de valgisation par ouverture interne constitue une indication de choix dans le traitement de la gonarthrose notamment chez les sujets jeunes. Grâce au planning préopératoire, cette technique permet une correction précise. L'ostéosynthèse joue un rôle important dans le maintient de la correction obtenue. Elle doit être stable, peu encombrante et permettant une chirurgie la moins invasive possible. L'ostéotomie tibiale d'addition par ouverture interne, dans notre contexte est la technique la plus adaptée car elle préserve le stock osseux métaphysaire et n'induit pas de cal vicieux extraarticulaire. La reprise ultérieure par prothèse totale de genou est relativement plus facile. Les améliorations techniques ont considérablement simplifiés l'intervention et ont élargi ses indications.

## Méthodes

Il s'agit d′une étude rétrospective comptant 38 genoux chez 28 patients âgés de plus de 40ans, colligés dans le service de traumato-orthopédie de l'hôpital Ibn Rochd de Casablanca entre 2002 et 2010. Pour réaliser cette étude, nous avons fait appel à: une fiche d'exploitation des dossiers, comportant les précisions suivantes: l’âge, sexe, profession; les antécédents pathologiques ou tares, les étiologies, le délai de consultation; examen clinique: douleur, marche, mobilité, stabilité, déformation, épanchement articulaire, état vasculo-nerveux des malades; l′arthrose fémoro-patellaire annexée à celle fémoro-tibiale interne selon Ahlbacke; le traitement instauré: type d′anesthésie, gestes associés, immobilisation, incidents, rééducation, complications immédiates et secondaires; bilan radiologique réalisé, déviation axiale, axe épiphysaire. Nous avons retenu pour cette étude les patients ayant un recul minimum de 2 ans (de 2 à 9 ans) avec un bilan radio-clinique complet.

## Résultats

38 genoux chez 28 patients âgés de plus de 40ans, avec une moyenne d′âge de 52ans, 18 femmes et 10 hommes, colligés dans le service de traumato-orthopédie de l'hôpital Ibn Rochd de Casablanca entre 2002 et 2010. Nette prédominance féminine: 64% Tous étaient autonomes et actifs; L’âge moyen est de 48 ans. (avec des extrêmes allant de 40 à 67ans) Le recul moyen était de 3,7 ans (de 2 à 9 ans) Le coté droit a été majoritairement atteint dans 72%, l′atteinte était bilatérale chez 10 patients qui ont été opérés à un intervalle de 14 mois en moyenne des deux genoux (10 à 35 mois); chez tous nos patients, on a soulevé un retard de consultation, moyennement évalué à 4ans (3 à 8 ans) Cliniquement, tous nos patients consultaient pour des gonalgies sur des genoux vierges de toute chirurgie, sans de laxité ni frontale, ni sagittale. Le genu varum était secondaire post-traumatique dans 8 cas et primitif dans 20. Le bilan radiologique préopératoire comportait dans tous les cas une incidence de face, de face en schuss, de profil à 30° de flexion, et un pangonogramme en appui bipodal, sur lequel a été mesuré l'angle HKA, l′axe mécanique fémoral et l′axe mécanique tibial. Les radiographies en postopératoire immédiat comportaient une incidence de face, de profil et un défilé fémoro-patellaire à 30°. Sur le plan radiologique, la gonarthrose fémoro-tibiale interne a été classée selon les stades d'Ahlback (Tableau 1): **Stade I**: pincement de l'interligne fémorotibial < 50%; **Stade II**: pincement de l'interligne fémorotibial > 50%; **Stade III**: Existence d'une cupule du plateau tibial dont la profondeur est < 5 mm; Stade IV: Existence d'une cupule du plateau tibial de profondeur entre 5 et 10 mm; Stade V: L'usure du plateau tibial est > 10 mm associée à une subluxation latérale du tibia L′arthrose fémoro-patellaire n′a été notée que dans seul cas.

**Tableau 1:** Classification de l'arthrose F-T selon Ahlback

Arthrose fémoro tibiale	Nombre de cas	Pourcentage%
Stade I	26	68,4
Stade II	10	26,3
Stade III	2	5,2
Stade IV	0	0

**Figure 1:**
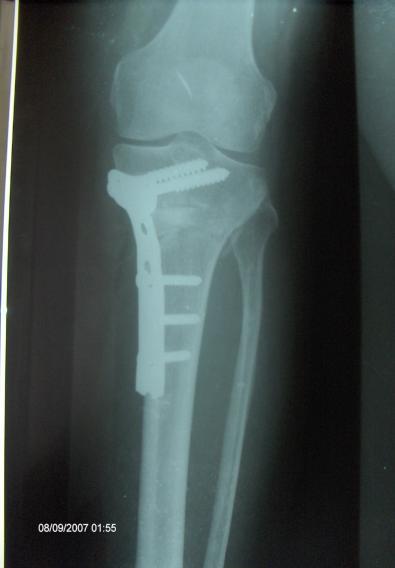
Radiographie du genou face objectivant une ostéotomie d'addition interne avec cale de ciment ostéosynthésée par plaque en L à 3 mois de recul

**Figure 2:**
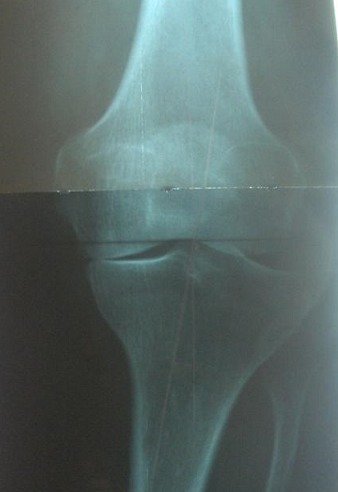
Radiographie du genou gauche en charge chez un patient présentant un genu varum de 15° avec pincement de l'interligne avec une arthrose du compartiment interne stade II d'Ahlback

Tous stades d'Ahlback confondus, la moyenne de déviation angulaire (le varum global moyen) était de 11,3°. L'ostéotomie est fixée par une plaque console en L inversé avec comblement partiel de l′ouverture de l′ostéotomie par interposition d′une cale cimentée; la cale reposant sur la plaque et occupant au maximum les deux tiers internes de la pyramide d′ostéotomie, s′arrêtant ainsi à distance de la charnière latérale. Le drainage étant systématique retiré à J2 post-op. (Figure 1, Figure 2) La rééducation active aidée du genou a débuté dès le lendemain, au service puis poursuivie dans un service de rééducation à l'hôpital. L'appui partiel était permis à partir de la 6^ème^ semaine en moyenne dans la plupart des cas. L'appui total était permis à partir de la 9 ème semaine en moyenne avec des extrêmes entre la 8^ème^ et la 10 ^ème^ semaine. La consolidation a été obtenue en moyenne au bout de 02 mois, il n'y a pas eu de pseudarthrose, la douleur s′étant amendée au terme du protocole de rééducation, 3 cas de sepsis superficiel ayant favorablement évolué sous antibiothérapie, 4 cas d′hématomes spontanément résorbés, et un seul cas de phlébite jugulée par anticoagulation curative; Aucun cas de raideur ou de déplacement secondaire n′a été noté. Les résultats fonctionnels ont été évalués selon la classification du groupe Guepar, ils étaient excellents et très bons dans 86%, moyens et mauvais dans 14% (Tableau 2).

**Tableau 2:** Classification du groupe Guépar

Résultat global	Douleur	Mobilité en flexion	Instabilité
Très bon	Aucune	>110°	Aucune
Bon	Modérée	90-109°	Modérée
Moyen	Importante	60-89°	Importante
Mauvais	Permanente	<60°	Permanente

## Discussion

Le traitement chirurgical de la gonarthrose unicompartimentale interne sur genu varum repose lorsque l'interligne est horizontal sur une ostéotomie tibiale de valgisation dont le principe est largement accepté puisque quelque soit la technique employée, elle entraîne régulièrement une amélioration de la symptomatologie fonctionnelle [1–7]. L'ostéotomie d'ouverture interne qui est prônée par plusieurs auteurs [8–14] est caractérisée par sa simplicité et sa sûreté par rapport aux ostéotomies curviplanes [15]. Elle a l'avantage d’éviter les complications neurologiques du nerf sciatique poplité externe et les ostéotomies du péroné nécessaires dans les ostéotomies par soustraction externe. Il n'y a pas aussi de risque de syndrome de loge dans cette technique [16], et évite aussi les complications septiques articulaires rencontrées, dans les ostéotomies en dôme maintenues par fixateur externe [17]. Elle a l'avantage de remettre en tension l'appareil ligamentaire interne. Cette remise en tension trouve toute son utilité sur les genoux instables avec désaxation en varus réductible complètement par la mise en valgus avec bâillement de l'interligne interne [18–21]. Elle permet également une ostéotomie itérative d'ouverture en cas de récidive du varus fémorotibial. Celle-ci est possible en cas de l'absence de chondrocalcinose et de dégradation fémoro-tibiale externe ou fémoropatellaire [22]. Il est possible aussi par cette technique de corriger de grandes déformations sans induire de cal vicieux métaphysaire [23, 24]. Néanmoins, il a été rapporté dans la littérature, que l'ostéotomie d'ouverture entraîne un abaissement de la rotule et déplace en dehors la tubérosité tibiale antérieure, mais l’étude à long terme de ces modifications n'a pas montré d'effets secondaires notables [7, 10, 11, 16, 25]. Cependant, d'après Hernigou [11], si elle est effectuée correctement avec une ouverture médiane et aussi postérieure, elle n'abaisse pas la rotule, elle peut même si la cale est très postérieure, diminuer la fuite postérieure du plateau tibial. Parmi les autres inconvénients, il y a aussi l'allongement du membre [20], fracture du plateau tibia externe et la pseudarthrose [15, 20]. Aucune des complications sus-citées n′a été rapportée dans notre série jusqu′à présent, mais vu nos résultats et le recul acquis, il nous est permis de conforter et d′appuyer les données apportées par les autres séries par la nôtre.

Par contre, les critiques apportées à cette technique, il y a 2 décennies, étaient le prélèvement de greffons iliaques et la morbidité associées, la remise en appui tardive [9, 12, 20] et la relative imprécision des mesures et de la correction effectuée contrairement à la fermeture externe qui permet des corrections dans plusieurs plans, ne nécessite pas de greffe osseuse, évite le bâillement osseux rencontré dans l'ouverture interne, dont le risque majeur est la non consolidation et permet aussi une mise en charge rapide [17, 20, 23]. La mise en place de greffons corticospongieux iliaques dans l'ouverture présente deux inconvénients non négligeables: une perte de correction qui peut survenir par résorption des greffons pendant la phase de consolidation; la morbidité liée à la prise du greffon iliaque: douleur post-opératoire, gêne au port de certains vêtements, infections, fractures, névralgie d'origine fémoro-cutanée, pseudarthrose [10]. Pour toutes raisons, qu′a été justifié notre recours à l'utilisation d'une cale de ciment selon la technique de Goutallier [10] qui permet de déterminer et de maintenir la hauteur de l'ouverture qui se fait autour de la charnière osseuse externe. On utilise dans le service, un polymère à base de polyméthyl métacrylate qui possède un antibiotique additif (la gentalline). Cette cale est confectionnée extemporanément après la mesure de la hauteur de l'ouverture grâce à un artifice de 2 écarteurs de Farabeuf avant l'incision permettant un gain de temps non négligeable sur la durée de pose du garrot pneumatique. Le traitement de l'arthrose fémoro-tibiale interne sur genu varum, par ostéotomie tibiale de valgisation, est parfaitement codifié à l'heure actuelle. Néanmoins, de nombreux auteurs soulignent la détérioration des résultats, à mesure que le recul augmente. Ainsi, l'ostéotomie tibiale de valgisation donne d'excellents résultats; Toutefois des facteurs de mauvais résultat existent néanmoins: usure du compartiment opposé à la déformation (chondropathie externe sur arthrose interne et déformation en genuvarum); usure de l'articulation fémoro-patellaire; instabilité chronique du genou; insuffisance ligamentaire due à l'ancienneté de l'usure et de la déformation; raideur du genou (surtout si flessum); grand âge > 60 ans L’âge n'est pas un facteur limitant dans le choix thérapeutique et l'ostéotomie tibiale de valgisation garde une place prépondérante dans l'arsenal thérapeutique de la gonarthrose sur genu-varum et permet d'assurer une amélioration clinique à court et moyen terme. Toutefois, le seul garant d'un bon résultat durable est l'analyse minutieuse de la gêne clinique fonctionnelle, des paramètres radiologiques et enfin une technique adéquate une fois l'indication d'une ostéotomie tibiale de valgisation retenue.

## Conclusion

L'ostéotomie tibiale haute de valgisation par addition interne utilisant une cale de ciment et sur genu varum évite la morbidité des greffons iliaques sans entraîner de complication particulière et sans allonger les délais de consolidation radiologique et de reprise de l'appui. Plus facile dans sa réalisation, raccourcissant le temps de l'intervention et les suites opératoires, elle démontre sa fiabilité et sa responsabilité quant à la précision de la correction et sa stabilité dans le temps, garant d'un résultat fonctionnel à long terme. Elle ne compromet pas la réalisation d'une arthroplastie totale du genou ultérieure car le stock osseux métaphysaire est préservé, elle n'induit pas de cal vicieux métaphysaire et l'ablation du matériel d'ostéosynthèse et de la cale en ciment ne présente aucune difficulté. Plusieurs facteurs concourent à la réussite de cette technique: Un âge inférieur à 60 ans, une mobilité préopératoire satisfaisante avec une flexion de plus de 90° et un flessum de moins de 5°, une bonne stabilité du genou, une arthrose fémoro-tibiale interne débutante de stade I et II d'Ahlback et une déformation en varus de moins de 15°. Mais une correction optimale entre 3° et 6° reste indépendamment de la technique utilisée, le principal facteur pronostic dont dépendent les résultats thérapeutiques surtout à long terme. L'ostéotomie tibiale de valgisation par addition interne est une intervention de pratique courante qui garde une large place dans le traitement de l'arthrose fémorotibiale interne et représente à l'heure actuelle pour nombreux auteurs comme pour nous également une technique prépondérante et une indication de choix chez les sujets jeunes actifs.
